# Development of Organic-Inorganic Hybrid Optical Gas Sensors for the Non-Invasive Monitoring of Pathogenic Bacteria

**DOI:** 10.3390/s18103189

**Published:** 2018-09-21

**Authors:** Sumana Kladsomboon, Chadinee Thippakorn, Thara Seesaard

**Affiliations:** 1 Department of Radiological Technology, Faculty of Medical Technology, Mahidol University, Phutthamonthon, Nakhon Pathom 73170, Thailand; sumana.kla@mahidol.edu; 2 Center for Research and Innovation, Faculty of Medical Technology, Mahidol University, Phutthamonthon, Nakhon Pathom 73170, Thailand; chadinee.thi@mahidol.ac.th; 3 Department of Physics, Faculty of Science and Technology, Kanchanaburi Rajabhat University, Kanchanaburi 71000, Thailand

**Keywords:** artificial nose system, metallo-porphyrins, metallo-phthalocyanine, optical gas sensor, bacterial identification

## Abstract

Hybrid optical gas sensors, based on different organic and inorganic materials, are proposed in this paper, with the aim of using them as optical artificial nose systems. Three types of organic and inorganic dyes, namely zinc-porphyrin, manganese-porphyrin, and zinc-phthalocyanine, were used as gas sensing materials to fabricate a thin-film coating on glass substrates. The performance of the gas sensor was enhanced by a thermal treatment process. The optical absorption spectra and morphological structure of the sensing films were confirmed by UV-Vis spectrophotometer and atomic force microscope, respectively. The optical gas sensors were tested with various volatile compounds, such as acetic acid, acetone, ammonia, ethanol, ethyl acetate, and formaldehyde, which are commonly found to be released during the growth of bacteria. These sensors were used to detect and discriminate between the bacterial odors of three pathogenic species (*Staphylococcus aureus, Escherichia coli and Pseudomonas aeruginosa*) grown in Luria-Bertani medium. Based on a pattern recognition (PARC) technique, we showed that the proposed hybrid optical gas sensors can discriminate among the three pathogenic bacterial odors and that the volatile organic compound (VOC) odor pattern of each bacterium was dependent on the phase of bacterial growth.

## 1. Introduction

In the medical care sector, many researchers are striving to exploit the chemistry of bacteria for early detection and surveillance of infectious diseases. Most infections in patients are caused by one of several types of pathogenic bacteria that can be found in the environment: *Staphylococcus aureus*, *Escherichia coli*, *Pseudomonas aeruginosa,* and so on. Many serious infections, such as pneumonia [1], meningitis [2], osteomyelitis [3], toxic shock syndrome [4], bacteremia, and sepsis [5], are often caused by the gram positive bacteria, *S*. *aureus*. In contrast, two types of gram negative bacteria are prominent for other reasons. *P*. *aeruginosa* is often the cause of infections in hospitalized patients and has a high rate of resistance to a variety of antibiotics [6]. *E*. *coli*, which may produce a Shiga toxin, can cause severe illness in humans and food poisoning [7]. Many bacterial species are present in both indoor and outdoor environments and can enter the human body through a break in the skin or mucosa. It is therefore absolutely necessary to determine the presence or absence of pathogenic bacteria prior to starting antibiotics. In general, conventional culture methods used to identify species of bacteria take at least 12–48 h [8]. Therefore, development of a simple non-invasive method for early detection and identification of bacteria has played a key role in the advancement of medical screening and surveillance. During recent decades there has been increasing interest in developing alternative methods for identification of individual strains of bacteria by the use of specific volatile organic compounds (VOCs) analysis [9]. Several species of bacteria produce unique VOC profiles and may generate characteristic odors [10]. Based on gas chromatography-mass spectrometry (GC-MS) analyses, ammonia is the major VOC from *P*. *aeruginosa* and *S. aureus* [11,12], while methanol [13], 1-propanol [9], 1-butanol [14], and indole [9] are associated with *E*. *coli*. Moreover, all bacteria generally produce different amounts of formaldehyde and ethanol vapors during their growing period. Although GC-MS analysis is recognized as a reliable procedure for bacterial identification, there are some problems and limitations that stand in its way, such as high cost and complicated processes. Because of these restrictions, many researchers in the field of sensor technology are trying to develop new techniques for classifying bacteria. Such methods, known as electronic noses, will make operations faster and easier than ever before [15]. Artificial nose systems are used to convert chemical information regarding volatile molecules into electrical signals. These consist of three components, each with a specific duty: (i) chemical vapor detection with gas sensor array platforms; (ii) signal processing; and (iii) pattern recognition [16,17,18]. Pattern recognition methods and algorithms are commonly employed in reusable, sensor-based VOC detection with real samples that can be directly analyzed without complicated sample preparation [19]. Therefore, these methods will not only be helpful in sensor array research for detection of odor signatures for screening and early identification, but also have great potential for clinical application without special laboratory equipment [20].

The optical gas sensor is one of many categories of gas sensor technology that can be investigated scientifically based on a common analytical instrument such as the UV-Vis spectrophotometer [21]. For example, an optical gas sensor combined with an electronic nose system is capable of monitoring for food spoilage based on the VOCs production from specific bacterial activity [22]. Selection of optical materials for sensor design and fabrication depends on a variety of factors such as the strength of reaction between the sensing material and one or more of the target odor compounds. Other factors are the color change which occurs during the gas absorption and the stability of sensors [23]. In general, optical gas sensors are usually fabricated from two types of organic dyes, metallo-porphyrins and metallo-phthalocyanine, which are both antibacterial compounds [24,25]. These organic dyes have markedly extended π-electron systems and exhibit stability at room temperature [26]. Modifications of the basic skeletons and central metal atom of the sensing materials lead to their remarkably high sensitivity to VOCs [27]. The sensitive area of the sensor is frequently observed in the near Q and B band regions of the light absorption spectrum [28]. Based on quantum mechanical (QM) calculations, there is a transfer of electric charge between the analyte gas and the central metal atom of the organic dye that indicates a potential change of their optical spectrum [29]. The metallo-porphyrins and metallo-phthalocyanine mentioned above were discovered to be excellent sensing materials for optical gas sensors based on changes in optical absorption of amorphous thin films. Specifically, zinc-porphyrin [30], manganese-porphyrin [31], and zinc-phthalocyanine [31,32] are used as common gas sensors for detection of various types of VOCs such as alcohol, acid, ketone, amine, and aldehyde. In terms of gas sensing properties, surface structure modification of sensing materials has the potential to enhance the sensing properties of gas sensors. Surface morphology of the films has changed greatly due to the thermal treatment process [33]. This process affects optical properties, such as absorption spectrum, that are related to the sensitivity of the sensors. For example, optimization of the grain size of thin-surface-layers can be expanded to provide greater surface area of the gas sensor for interaction with the absorbed analyte molecules [34]. Such research has yielded many useful fabrication processes for development of better optical gas sensors. However, there is no published work related to the fabrication of organic-inorganic hybrid, optical gas sensors with three different types of dye (zinc-porphyrin, manganese-porphyrin, and zinc-phthalocyanine). Therefore, our research work extended the functionality of optical gas sensors that generate a wide range of absorption spectra. Moreover, the optical gas sensors we fabricated have many advantages such as increasing the active sites for gas detection to improve the gas sensor response. Thus, the emergence of a hybrid optical gas sensor based on three types of dye will open new frontiers in pathogenic bacteria odor monitoring.

Accordingly, the aim of this study was to assess the feasibility and performance of optical gas sensors based on an artificial nose system for the rapid detection and classification of bacterial pathogens, specifically *P*. *aeruginosa*, *E*. *coli* and *S*. *aureus*. The optical gas sensors were fabricated from three organic-inorganic hybrid materials. Sensitivity of these sensors was enhanced by thermal treatment. Statistical methods, such principal components analysis (PCA), were used to identify the pattern recognition and classification of VOCs from the three different bacterial species grown in culture medium. In addition, we evaluated the accuracy in each change of signal-response using the *p*-value method (Hypothesis testing). Results of this study may provide data to consider in choosing specific optical gas sensors that are appropriate for detection of bacterial odors in order to protect the public from bacterial contamination of food and drink in the manufacturing industry and in the environment.

## 2. Materials and Methods

### 2.1. Optical Gas Sensor Fabrication

Zinc-5,10,15,20-tetra-phenyl-21H,23H-porphyrin (ZnTPP), zinc-2,9,16,23-tetra-tert-butyl-29H, 31H-phthalocyanine (ZnTTBPc), and manganese (III)-5,10,15,20-tetraphenyl-21H,23H-porphyrin chloride (MnTPPCl) were purchased from Sigma-Aldrich (St. Louis, MO, USA). The ZnTPP, ZnTTBPc, and MnTPPCl were dissolved in chloroform at the concentrations 5, 10 and 15 mg/mL, respectively. Then, equal volumes of the three solutions were mixed together. Then, 0.5 mL of the mixed solution was deposited on clean glass substrates, using the spin coating method at a speed of 1000 rpm for 30 s. Finally, the sensing films were enhanced by thermal treatment for 30 min at 150 °C under air atmosphere.

### 2.2. Characterization of the Hybrid Optical Gas Sensor

**Figure 1:**
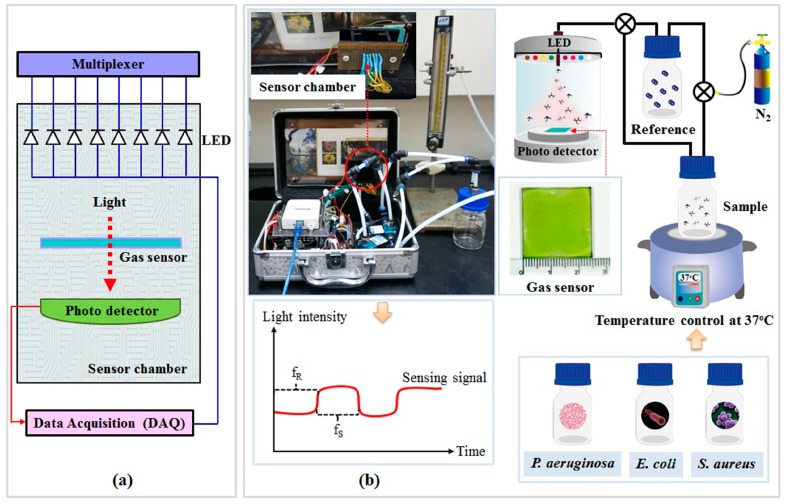
Schematic diagram of the optical artificial nose system, including (**a**) measurement/controller circuit section and (**b**) odor delivery section.

Change of optical absorption spectra of gas sensors for both the non-treated and treated sensing films were investigated with a UV-Vis spectrophotometer (Shimadzu UV-2450, Tokyo, Japan). The hybrid optical gas sensors were placed into a stable VOC atmosphere chamber to observe their sensitivity to six vapors: acetic acid, acetone, ammonia, ethanol, ethyl acetate, and formaldehyde. The absorption index of gas sensors when tested along with each VOC were recorded in the visible spectral range of 300–700 nm. Moreover, the optical gas sensors’ sensitivity under dynamic gas flow conditions was investigated by an optical artificial nose system (see Figure 1). All tested VOC solutions were prepared at the concentration of 10% (volume/volume) in water. Once a VOC sample was incorporated into a sample bottle, the operating temperature was adjusted for each VOC sample to control for the same evaporating pressure of each VOC through the sensor chamber. In addition, a nitrogen carrier system was installed inside the system to spread the VOC’s vapor evenly across the bottle, delivering volatile vapors to the sensor chamber. Two cases of the non-treated and treated gas sensors were consecutively exposed to the VOCs for 2 min and N_2_ for 2 min at room temperature. Lastly, structure and morphology of the gas sensor were measured by atomic force microscopy (Agilent 5500 AFM, Agilent Technologies, Chandler, AZ, USA).

### 2.3. Optical Artificial Nose System

The optical artificial nose system for odor detection was composed of a hybrid optical sensor and data acquisition algorithm. The schematic diagram of the in-house artificial nose system is shown in Figure 1. This system involves two main elements: measurement/controller circuit section and odor delivery section. A National Instruments data acquisition (Ni-DAQ) USB-6008 card with LabVIEW was chosen as the measurement and the control device (analog input and digital output), see Figure 1a. Low cost commercial LED lamps were used to create an artificial light source. Each channel of the analog multiplexer selected one of several input signals from individual LED lamps and forwarded the selected input into a single line to the output signal frequency of each LED lamp. The optical transducer (CMOS photo-detector), which converted light into an electrical quantity, was chosen to collect the light intensity transmitted through the optical gas sensor. This photo-detector was the color light-to-frequency converter module (TCS230) from Texas Advanced Optoelectronic Solutions Company (Plano, TX, USA). The output data was constructed as a square wave with frequency directly proportional to light intensity. Wavelengths corresponding to red, yellow, green, pink, blue, and violet LEDs were centered at 638, 587, 537, 472, 457, and 399 nm, respectively, while white and infrared LEDs represented the broad spectrum light around 450–700 and 700–1 × 10^6^ nm, respectively. The intensity of light transmitted through the optical gas sensor was detected from the photon frequency (Hz) that interacted directly with the photo-detector during the dynamic gas flow measurement, see Figure 1b. An array of optical gas sensors was generated with LED lights of eight colors (infrared, red, yellow, green, violet, pink, blue, and white). Nitrogen gas (N_2_) was used as the pure carrier (reference) gas, delivering odors to the sensor chamber. The flow rate of the N_2_ gas was controlled by a mass flow meter at a constant rate of 700 mL/min. Measurement of the dynamic gas flow system was performed by switching between the sample gases for 2 min and the N_2_ for 2 min. This process was repeated for 5 cycles. The gas sensing response (S) from the eight sensors was the difference between the maximum peak (signal frequency from the sample odor) and the baseline (signal frequency from the reference gas). These responses were used as the input data for pattern recognition by principal component analysis (PCA). The gas sensing response (S) was defined as the light intensity change in the frequency during the presence of dynamic gas flow measurements as follows (Equation (1)):

(1) $$S=\left(\frac{f_{S}-f_{R}}{f_{R}}\right)\times 100=\left(\frac{\Delta f}{f_{R}}\right)\times 100\;$$

where f_R_ is the initial frequency of each optical wavelength without the sample vapor (baseline frequency) and f_S_ is the frequency when exposed to the testing gas vapor, see in Figure 1b. The gas sensing response (S) can be calculated from a differential comparison between the initial frequency of each optical wavelength (baseline frequency or f_R_) and the frequency when exposed to the testing gas vapor (f_S_). Therefore, spectral sensitivity can be represented as a percentage of the changes in the frequency signal relative to the initial frequency of each optical wavelength.

### 2.4. Bacterial Cultures

Three standard strains of bacteria were selected for this study. *Staphylococcus aureus* *subsp*. *aureus* (ATCC 29213), *Escherichia coli* (ATCC 25244), and *Pseudomonas aeruginosa* (ATCC 27853) were obtained from the Faculty of Medical Technology, Mahidol University, Thailand. They were cultured in sterile nutrient medium and incubated at the optimum temperature for growth. Briefly, batch cultivation was carried out in Luria-Bertani (LB) medium at 37 °C. The composition of the LB broth per liter was Tryptone (Difco Laboratories) 10 g, Yeast extract (Difco) 5 g, and NaCl 5 g. Medium was made using distilled water and adjusted to within a pH range of 7.2–7.4 using diluted solutions of NaOH and then autoclaved under standard conditions of temperature and pressure (121 °C at 15 psi) for 15 min (followed the Miller’s formula). Single bacterial colonies from LB agar plates were inoculated into 5 mL of LB broth and maintained at 37 °C for 9 h in a shaking incubator (180 rpm). After incubation, bacterial growth was characterized by a cloudy media. The concentration of bacteria in medium was obtained by a measurement of the optical density at 600 nm (OD600) of the culture suspensions with a UV-Visible spectrophotometer. The three different types of initiating bacterial cultures were adjusted until the OD600 was equal to 0.3. Then, each bacterial culture was inoculated into 25 mL of LB broth to control the initial bacterial counts in the sample bottles for odor detection.

Bacterial cultures were maintained at a constant incubation temperature (IT) of 37 °C. Culture samples were removed at intervals and the numbers of viable bacteria were counted (the increasing turbidity assessed at OD600). A logarithmic growth curve of each bacterial strain was plotted. Finally, the OD600 value and gas sensing response of each bacterial culture were collected and recorded every 3 h.

## 3. Results and Discussion

### 3.1. Optical Gas Sensor Characterization

**Figure 2:**
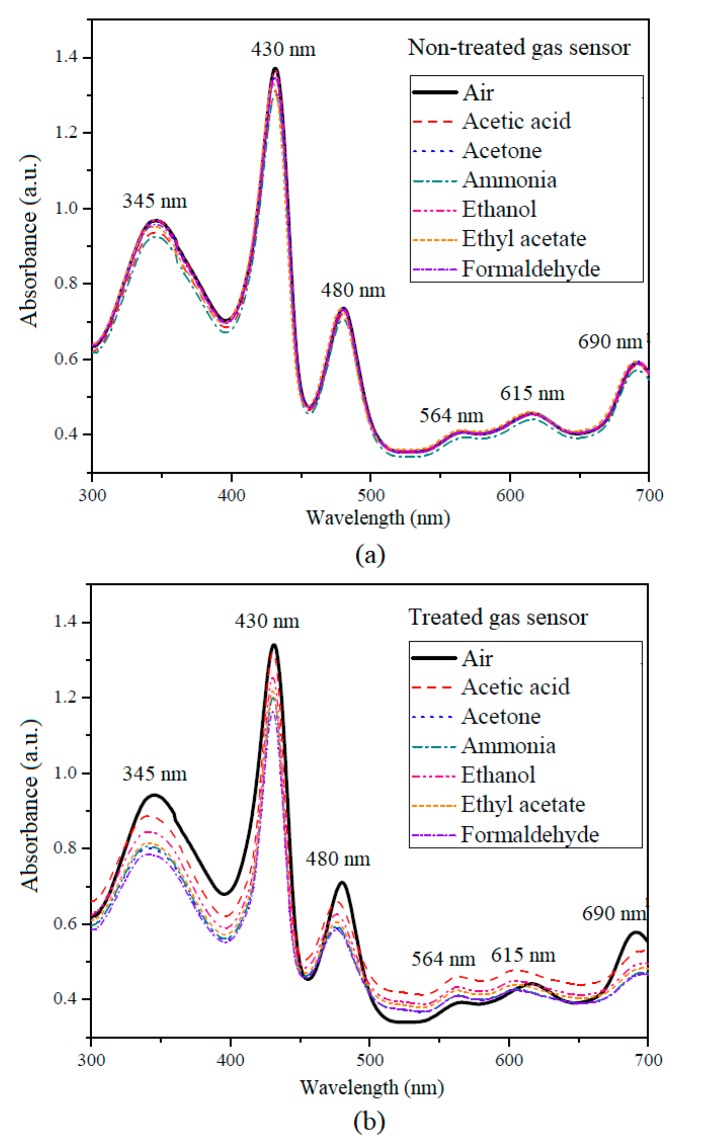
UV-Visible absorption spectra changes of (**a**) non-treated and (**b**) thermally treated gas sensors with volatile organic compound (VOC) exposure.

In this work, the hybrid optical gas sensor was fabricated by employing metal-free organic-inorganic dyes, namely ZnTPP, ZnTTBPc, and MnTPPCl. Effects of thermal treatment on the structure and the sensitivity properties of the optical gas sensor were investigated by UV-Vis spectroscopy. Figure 2 shows the changes in the absorption spectrum of optical gas sensors with and without thermal treatment. Six volatile compounds (10% in water, acetic acid, acetone, ammonia, ethanol, ethyl acetate and formaldehyde) associated with bacterial metabolism and released during bacterial growth were selected for the testing of sensor performance [10,11,12,13,14]. In the case of the non-treated gas sensor, maximum absorptions centered at 345, 430, 480, 564, 615, and 690 nm (see Figure 2a). The Soret bands of ZnTTBPc, ZnTPP, and MnTPPCl are the absorption peaks at 345, 430, and 480 nm [30], respectively. The absorption bands observed in the 400–500 nm range are related to n-π* transitions of the lone nitrogen pair orbital of the macrocycle. The peaks at 564 and 615 nm are the Q bands of porphyrin compounds, while the peak at 690 nm is the Q band of the ZnTTBPc. These Q bands are related to the π-π* transitions of the porphyrin macrocycle ring [35]. The shift of the absorption spectra in both cases (with and without thermal treatment) of the gas sensors when exposed to VOs vapors were observed near the main peaks, namely 345, 430, 480, and 690 nm (see Figure 2a,b). These absorption spectral shifts of the gas sensors are related to the electron interchange between the analyte gas and π-π conjugated system of the porphyrin molecule that effects the electron density π-π* transitions [36]. Our results showed that the absorption spectra of the thermal-treated gas sensor had much stronger changes than did the non-treated sensor, especially at 345, 430, and 480 nm. Therefore, the absorption spectral changes of the Q and B bands of the gas sensor were calculated with special attention.

The changes in the absorption spectra of the optical gas sensors between the atmospheric N_2_ and the six volatile organic compounds representative of those released during bacterial growth were calculated using Equation (2) below.

(2) $$A_{W}^{S}-A_{W}^{R}\;$$

where $A_{W}^{S}$ is the absorption spectrum of the analyte gas at w wavelength and $A_{W}^{R}$ is the absorption spectrum of the reference gas at w wavelength.

**Figure 3:**
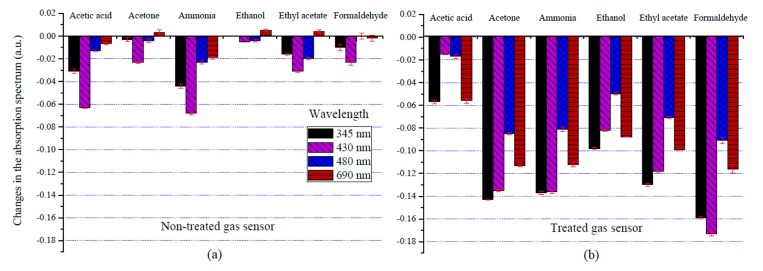
Changes in the absorption spectrum of (**a**) non-treated and (**b**) thermally treated gas sensors with different VOC profiles at wavelengths of 345, 430 480, and 690 nm.

Figure 3a shows the change in the absorption spectra of the non-treated gas sensor with six VOCs at 345, 430, 480, and 690 nm. Almost all absorption spectrums of the gas sensor were decreased after exposure to the VOC vapors. The highest absorbance value was observed at 430 nm, which is the B band of porphyrin. The sensitivity of the optical gas sensor can be calculated from the changes in the absorption spectrum values of thermal treatment compared with those of the non-thermal treatment (see in Figure 3a,b). It can be seen that the changes in the absorption spectrum of the thermally treated gas sensor were higher than that of the non-treated gas sensor for all wavelengths.

Wavelengths of 345 and 430 nm were associated with higher responses than at other wavelengths, particularly in the response patterns of the six VOCs measured by UV-Visible spectroscopy. Moreover, the changes in the absorption spectra of the treated gas sensor with the VOCs tested were found to peak at approximately 430 nm, especially with formaldehyde vapor. In addition, the electric charge transferred between the analyte gas and the central metal atom of the organic dye during the interaction process can be explained by the quantum mechanical (QM) calculations based on density functional theory (DFT). The electric charge of dye molecule was significantly changed after chemisorption of the gas molecule at the central metal atom [30]. Subsequently, this charge transport path depends on the position of dye molecule on the substrate [29]. Thus, this calculation indicates that electric charge transfer has the potential to change the optical spectrum of organic dye.

**Figure 4:**
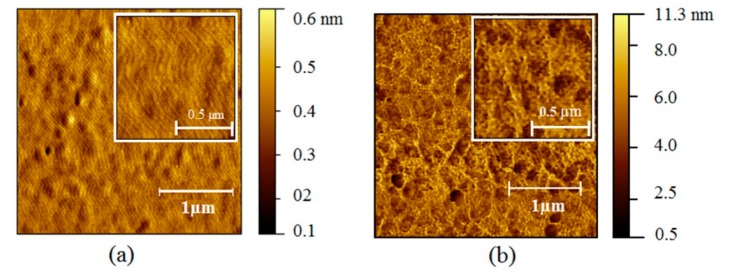
Atomic force microscopy (AFM) images (1 μm) of (**a**) non-treated and (**b**) thermally treated gas sensors.

The surface morphology of non-treated and treated gas sensors were characterized by using atomic force microscopy (AFM) as shown in Figure 4. The surface roughness of thin-film gas sensors for non-thermal treated and thermal treated film were 0.494 and 0.961 nm, respectively. Thus, the thermal treatment process can promote changes from a smooth surface to a knobbed surface [33]. Moreover, the average hole size of a thermal treated gas sensor was 0.1 µm, while the hole sizes of a non-treated gas sensor cannot be evaluated due to the smooth surface. It was evident that the surface morphology changed upon thermal treatment, and that surface roughness increased. This type of modified surface is associated with optimized trace gas detection due to shifts in the absorption spectra of the gas sensors [34], as can be seen in the bar graph (Figure 3) and the AFM images (Figure 4). Moreover, the AFM results in Figure 4 indicate that the thermal treatment has an effect on the structure and surface area on gas sensing films, leading to an increase of surface roughness. According to existing research, the temperature used in thin film preparation affects the morphology of the film’s surface [33]. Therefore, increasing the surface area of films generally enhances the rate of a chemical vapor reaction [34], which leads to the significant increase in the sensor’s response, as shown in Figure 3.

**Figure 5:**
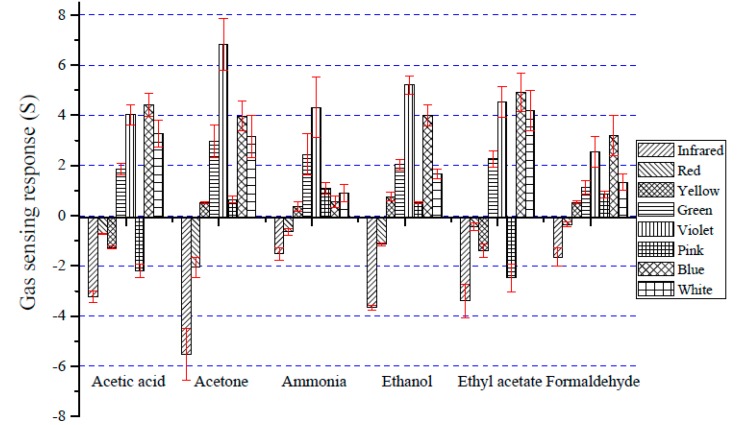
The gas sensing response (S) of thermally treated gas sensors toward VOCs in water (10%, volume/volume) under dynamic gas flow conditions.

Figure 5 shows a comparison of the gas sensing response (S) of the thermally treated gas sensor when exposed to different VOCs (10% in water) under dynamic gas flow conditions. An in-house optical-based artificial nose system was developed to study the performance of thin-film sensing materials. The gas sensing response (S) was calculated from the light intensity change between the sensor signal without sample vapor (baseline light intensity, f_R_) and the signal when exposed to the test vapor (f_S_) (see Equation (1)).

An array of eight optical gas sensors was generated from the different LED light sources: infrared, red, yellow, green, violet, pink, blue, and white. The sensitivity of gas sensors depends on the LED light source and the types of VOCs. The sensing signals from the yellow, green, violet, blue, and white LEDs showed positive S values, while the infrared, red, yellow, and pink LEDs showed negative S values. These results showed that light intensity changed when the sensors were placed in the vapor flow of the sampling system. Excellent sensing behavior was found in the case of the violet, blue, infrared, and white LEDs for all VOCs, demonstrating that the most active sites for sensing signals were at 399 nm, 457 nm, the infrared region, and broad spectrum light. The highest sensing response to the gases was found with acetone vapor and the violet LED. The gas sensor had distinct response patterns with different gases, which represented the odors that are released during bacterial growth. Therefore, the thermally treated gas sensor is proposed for bacterial identification.

### 3.2. Bacterial Growth and Pattern Analysis

**Figure 6:**
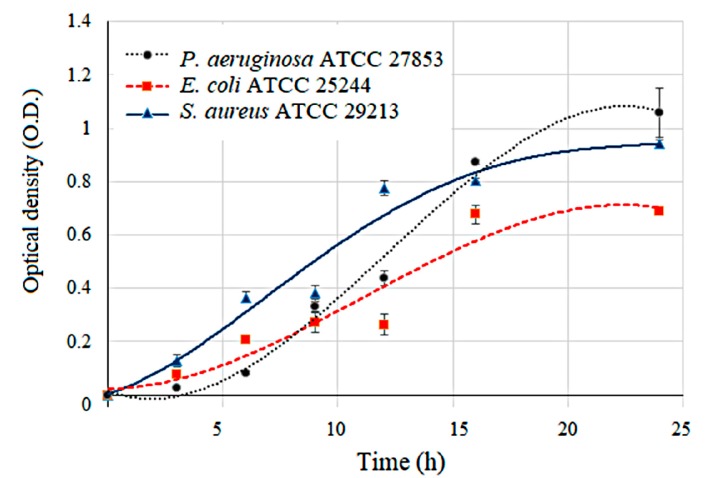
The optical density at 600 nm (OD600) was plotted versus time for individual pathogens in culture over an incubation period of 24 h. The growth curves were distinct for each pathogen. Blue triangle, red square, and black circle represent the data points used to construct the growth curves of *S. aureus*, *E. coli,* and *P. aeruginosa*, with R-squared (R^2^) statistics of 0.9628, 0.9214 and 0.9881, respectively.

Bacterial populations were quantitated periodically, and the number of viable bacteria was plotted on a log graph against time. This gives a bacterial growth characteristic which is known as the growth curve or growth cycle. Here, the growth of each bacterial strain in liquid media was investigated by observing the OD600 as shown in Figure 6. The absorption of this media was investigated (in triplicate) at the wavelength of 600 nm. An exponential growth phase was observed during the 24-h incubation. There were different growth characteristics for the three pathogens, and each had a high R-squared (R^2^) value. The growth rate of *P. aeruginosa* was compared with those of *S. aureus* and *E. coli* (R^2^ values of 0.9881, 0.9619, and 0.9214, respectively). The results indicated that the growth rate of E. coli was a bit less than those of *P. aeruginosa* and *S. aureus*. Moreover, the stationary phase (no further increase in the number of cells) of all three bacterial strains was observed after 16 h of incubation. The hybrid optical gas sensor was applied to investigate the relationship between the bacterial activity and VOC release.

**Figure 7:**
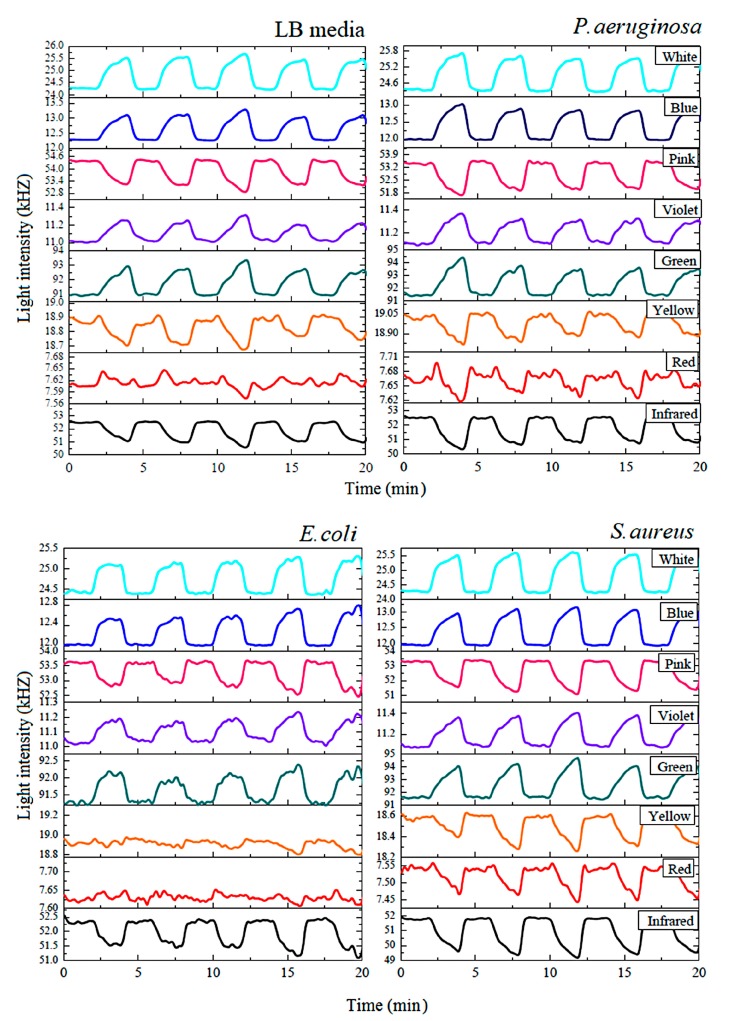
The gas sensing signal of the thermally treated gas sensor with Luria-Bertani (LB) media and the three types of bacteria as measured by the in-house, optical artificial nose.

The hybrid optical gas sensor was used to detect the volatiles produced by the three pathogenic bacteria and the pathogen-free LB media. Figure 7 illustrates the results of the optical gas sensor array signal from bacteria growing over a 9-h incubation. Nitrogen gas was used for gas-flushing to clean the chamber and also used as a carrier gas to deliver the odor to the sensor chamber. While conducting the measurement, all culture samples were maintained at a controlled temperature of about 37 °C. The sensorgram showed the level of the light intensity signal obtained from the optical sensor array. The design consisted of an eight-channel gas sensor, generated from eight LED light sources, namely infrared, red, yellow, green, blue, pink, violet, and white LED.

The measurement was performed by switching between the nitrogen gas for 2 min and the sample vapor for 2 min. This process was repeated five times for one measurement. The optical sensing signal was investigated in the form of photon frequency (kHz) and recorded every 4 s. Increased optical sensing signals were found with the green, blue, violet, and white LEDs, while decreased optical sensor signals were found with the infrared, red, yellow, and pink LEDs. The optical sensing signals of *P. aeruginosa* and *S. aureus* were found prominently displayed with all LED light sources, but *E. coli* was not observed with the red and yellow LEDs. Therefore, the optical sensing signal patterns of *P. aeruginosa* and *S. aureus* were different from those of *E. coli.* Even though the optical sensing signal patterns of *P. aeruginosa* were similar to those of *S. Aureus*, the optical sensing signal paths differed. Thus, optical gas sensors have a great potential to detect and classify these three pathogenic bacteria based on sensing signal pattern analysis.

**Figure 8:**
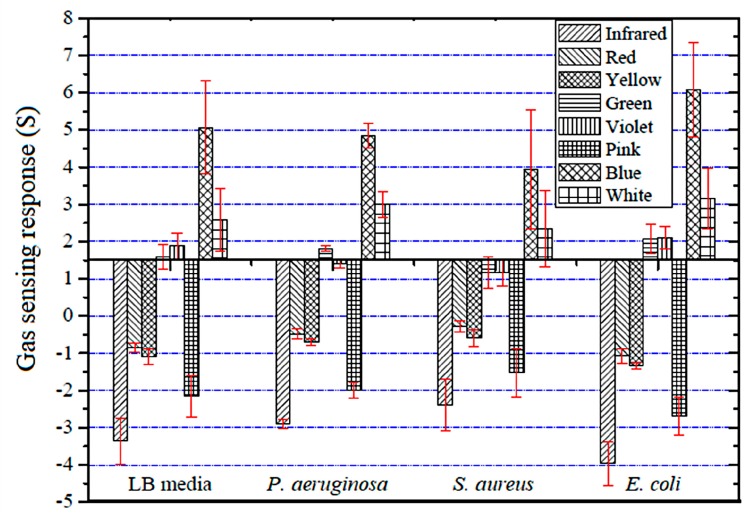
The gas sensing response (S) of the thermally treated optical gas sensor toward three species of bacteria in culture media after 9 h of incubation.

To evaluate the optical gas sensor’s potential for bacterial identification, we exposed it to the VOCs of three bacteria: *P. aeruginosa*, *S. aureus* and *E. coli*. The average percent change in the light intensity of optical sensors under the dynamic measurement system when exposed to volatile gas samples were calculated according to Equation (1). The results (Figure 8) indicated not only that blue LED showed the highest sensing response for the three types of bacteria, but also showed that the bacteria produce a wide range of VOCs in the differing patterns of exponential growth, under standard nutritional conditions (see Figure 6). These sensing responses of optical gas sensors are related to the amount and type of VOCs that are emitted during bacterial activity [37].

Additionally, hypothesis testing (*p*-value approach) was used to compare the sensor’s response to each of the three bacterial species and the LB media control at four different incubation times: 3, 6, 9, and 12 h. Their statistical significances (*p*-value in brackets) were all highly significant: infrared (0.00001), red (0.00000), yellow (0.00003), green (0.00008), violet (0.00058), pink (0.00000), blue (0.00004), and white (0.00021). This indicated that all optical gas sensors (with incubation time of 9 h) showed a significant difference in the sensor’s response between the three types of bacteria and LB media. However, the responses from the red sensor (0.36455) with an incubation time of 3 h were not significantly different among the bacteria samples and the LB media control.

An artificial nose technique was used to identify the physical features of odor patterns from the responses of all the sensors and to distinguish this pattern from a diverse range of smells (odor fingerprint of VOCs released by bacteria). Responses of optical gas sensors to odorants are generally considered as a first order time response. The first step in odor analysis is to flush a reference gas (N_2_) through the optical sensor to obtain a baseline. Then, the optical gas sensor is exposed to gas coming from the growing bacteria. This causes changes in the sensor’s output signal until a steady state is reached. Three major physical features were captured mathematically as min-max values, slope values, and integral area values and were selected for a feature extraction technique based on analysis of the covariance between the factors. Principal component analysis (PCA) is suitable for multi-dimensional datasets as in this work where data are collected from eight optical sensor arrays and multiple odors. The complex data set (with multiple dimensions) can be transformed using a matrix that represents general values in the form of a new data set (2 or 3 dimensions) [18]. In general, the PCA process consists of several steps which include: (i) preparing the raw data into a data matrix; (ii) scaling a data matrix by normalizing; (iii) calculating a covariance matrix; and (iv) rearranging the eigenvectors and eigenvalues. Then, the PCA result is obtained by matrix multiplication and transposition. The PCA results in this work were presented in the form of new principal components (PCs). The first principal component (PC1) contained more of the data variance of a data set than subsequent components (i.e., PC2 and PC3) [37].

**Figure 9:**
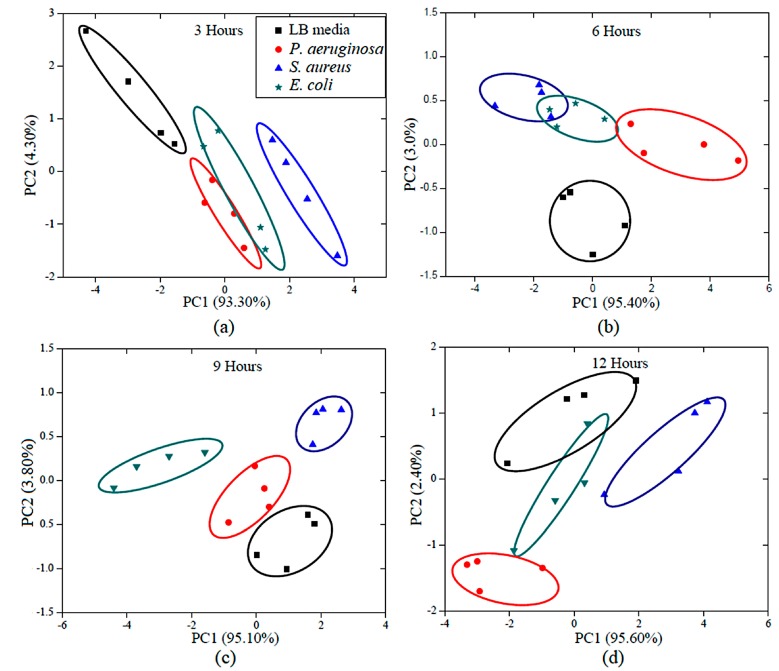
Schematic diagram of principal component analysis (PCA) of three types of pathogenic bacteria in medium with in-house artificial nose measurements at (**a**) 3 h, (**b**) 6 h, (**c**) 9 h and (**d**) 12 h.

Figure 9 shows the identification of three types of pathogenic bacteria and LB media in PCA plots. To see the effect of incubation period on growth and bacteria activity, a change in smell was investigated every 3 h. It was found that the pure LB medium, *P. aeruginosa*, *S. aureus*, and *E. coli* could be distinguished and grouped using the eclipses with 95% confidence. Figure 9a–d show the distinct clusters of pure LB media and the three bacteria samples, which are separated into four groups even at the earliest assessment (Figure 9a). After 9 h of incubation, discrimination between the different types of bacteria and LB media sample was seen (Figure 9c). PC1 accounts for the greatest variance (95.10%). Thus, it was found that the four data clusters are clearly separated on the PC2 axis, while the data points of the bacteria odors and LB media are scattered along the PC1 axis.

PCA results, with several incubation times, odor from three bacteria (*P. aeruginosa*, *E. coli*, and *S. aureus*), and the medium control, appeared close together on the PCA. This result agreed with the gas sensing response results from the VOCs emitted by the selected bacteria at different incubation times (see Figure 8). As a matter of fact, VOCs emitted by the different bacteria varied with stage of growth, which may be due to physical specificity, metabolic influences, or even time-dependent sampling [38]. It was found that the incubation time of 9 h gave each of three types of bacteria a unique pattern of odor (smell fingerprint), as their odor differed not only from each other, but also from the scent of the culture medium. These findings will pave the way for further development of specifically designed, optical gas sensors and artificial nose systems for bacterial odor detection which are both sensitive and specific.

## 4. Conclusions

The sensitivity and stability of hybrid optical gas sensors were enhanced by a thermal treatment process. The in-house optical artificial nose system based on a light emitting diode and photo detector was successful in investigating the characteristics of a new optical gas sensor within a dynamic gas flow system. The bacteria were successfully detected and identified by the PCA using an incubation time of 9 h. The results showed the feasibility of using an optical artificial nose system for real-time discrimination of bacteria odors to indicate the presence of different bacterial species and their phase of growth. The capability to detect various VOCs as related to the bacteria growth, especially acetic acid, acetone, ammonia, ethanol, ethyl acetate, and formaldehyde, has been demonstrated. Based on pattern recognition techniques for odor discrimination in optical gas sensor arrays, we have shown that the proposed hybrid optical sensing material combined with an artificial nose system can discriminate the odor of three bacteria species and allow non-invasive pathogenic bacterial monitoring.
